# Fructose-1, 6-bisphosphatase 1 interacts with NF-κB p65 to regulate breast tumorigenesis via PIM2 induced phosphorylation

**DOI:** 10.7150/thno.46861

**Published:** 2020-07-09

**Authors:** Chao Lu, Chune Ren, Tingting Yang, Yonghong Sun, Pengyun Qiao, Xue Han, Zhenhai Yu

**Affiliations:** 1 Department of Reproductive Medicine, Affiliated Hospital of Weifang Medical University, Weifang, Shandong Province, P.R. China.; 2 Department of Pathology, Affiliated Hospital of Weifang Medical University, Weifang, Shandong Province, P.R. China.

**Keywords:** PIM2, FBP1, phosphorylation, protein stability, tumor growth

## Abstract

**Rationale:** Fructose-1, 6-bisphosphatase 1 (FBP1), a rate-limiting enzyme in gluconeogenesis, was recently shown to be a tumor suppressor and could mediate the activities of multiple transcriptional factors via its non-canonical functions. However, the underlying mechanism of posttranscriptional modification on the non-canonical functions of FBP1 remains elusive.

**Methods:** We employed immunoaffinity purification to identify binding partner(s) and used co-immunoprecipitation to verify their interactions. Kinase reaction was used to confirm PIM2 could phosphorylate FBP1. Overexpression or knockdown proteins were used to assess the role in modulating p65 protein stability. Mechanistic analysis was involved in protein degradation and polyubiquitination assays. Nude mice and PIM2-knockout mice was used to study protein functions *in vitro* and *in vivo*.

**Results:** Here, we identified Proviral Insertion in Murine Lymphomas 2 (PIM2) as a new binding partner of FBP1, which could phosphorylate FBP1 on Ser144. Surprisingly, phosphorylated FBP1 Ser144 abrogated its interaction with NF-κB p65, promoting its protein stability through the CHIP-mediated proteasome pathway. Furthermore, phosphorylation of FBP1 on Ser144 increased p65 regulated PD-L1 expression. As a result, phosphorylation of FBP1 on Ser144 promoted breast tumor growth *in vitro* and *in vivo*. Moreover, the levels of PIM2 and pSer144-FBP1 proteins were positively correlated with each other in human breast cancer and PIM2 knockout mice.

**Conclusions:** Our findings revealed that phosphorylation noncanonical FBP1 by PIM2 was a novel regulator of NF-κB pathway, and highlights PIM2 inhibitors as breast cancer therapeutics.

## Introduction

Fructose-1, 6-biphosphatase 1 (FBP1) is a rate-limiting enzyme in gluconeogenesis, and functions as a negative regulator of the Warburg effect 1. As a tumor suppressor, FBP1 plays a crucial role in tumor progression in multiple cancers 2. Low expression of FBP1 is associated with tumorigenesis and poor prognosis in patients with breast, kidney, colon, pancreas, lung, stomach and liver cancers 3-8. In addition to its function as a metabolic enzyme, it also acts as a co-suppressor for multiple transcription factors to reduce downstream gene expression. For example, FBP1 can directly bind to hypoxia inducible factor 1α (HIF-1α), and regulates its transcriptional activity to oppose renal carcinoma progression 5. FBP1 high-regulation inhibits the activity of the WNT/β-catenin pathway and reduces the level of its downstream target genes 9. Moreover, FBP1 can directly destabilize c-MYC by disrupting the ERK-c-MYC axis, an action that has been shown to increase the sensitivity of pancreatic cancer cells to JQ1 10. Our previous study showed that FBP1 binds to NOTCH1 and enhances its ubiquitination, further leading to proteasomal degradation via the FBXW7 pathway 11. However, the underlying mechanisms of post-transcriptional modification for the non-canonical function of FBP1 remain elusive in breast cancer.

The PIM2 kinase belongs to a serine/threonine kinase family of three members (PIM1, PIM2 and PIM3), and was first identified as a proviral integration site for Moloney murine leukemia virus 2-induced T-cell lymphoma 12. These three kinases participate in several tumor progression factors, including cell cycle, survival, proliferation, migration, apoptosis, metabolism, and drug resistance 12. Unlike PIM1 and PIM3, PIM2 can be constitutively activated because it lacks a regulatory domain, and thus could be used to design drug targets 13. The function of PIM2 in cancer depends on its serine/threonine kinase activity, which can phosphorylate multiple substrates including p21, p27, NOTCH1, p65, BAD, AMPKα1, TSC2, PKM2, c-MYC, HK2 and HSF1 12, 14-18. Moreover, various special inhibitors, including JP11646, SMI-4a and SGI-1776, have been developed for PIM kinase activity and have been used for clinical treatment 19-21. Recently, we found that *PIM2* acts as an oncogene in breast cancer 15, 17, 22, but the underlying mechanism of its oncogene function remains largely unknown.

FBP1 has recently emerged as a broad co-repressor for multiple transcriptional factors via its non-canonical functions. In particular, we found that FBP1 inhibits NOTCH1 function via FBXW7-induced protein degradation 11. In this study, we uncovered that PIM2 can phosphorylate FBP1 at the Ser144 site, and decrease FBP1 binding to p65 independent of its enzyme activity. FBP1 phosphorylation by PIM2 promoted breast tumor growth and p65-induced PD-L1 expression, highlighting the role of PIM2-dependent FBP1 phosphorylation in breast tumor progression.

## Materials and Methods

### Cell culture

HEK293T, MCF-7 and MB231 cells were cultured in DMEM supplemented with 10% FBS. All cells were cultured at 37℃ supplied with 5% CO_2_.

### RNA interference

shRNAs were constructed into pLVX-shRNA1 vector. Viral packaging plasmids (pMD2.G and psPAX2) and shRNA plasmid were transfected to 293T cells by using lipofectamine 2000. After 24hr, virus culture medium was replaced with new DMEM containing 10% FBS. 48hr post transfection, the medium was collected and added to breast cancer cells added with polybrene. Breast cancer cells were harvested 48hr after puromycin selection. shRNA sequence information was provided in Table S2.

### Immunoprecipitation (IP) and GST pull-down assays

Cells were harvested and lysed with IP buffer (50mM Tris-HCl, pH 7.5, 150mM NaCl, and 0.5% NP40) with multiple protease inhibitors (Sigma-Aldrich). On ice for more than 30min and the lysate was centrifuged at 12000 rpm at 4℃ for 10 min. The supernatant was rocked overnight with protein A/G agarose beads and indicated antibodies under 4℃. The beads were washed at least 5 times using IP buffer, and then used for subsequent experiments. The indicated proteins were expressed in E.coli BL21 (DE3), and GST-pull down assay was performed as described previously 17.

### Phosphorylation assay

The kinase reaction buffer was performed as described previously 15, 16, 18. The reactions were subjected to Western blotting analysis.

### Putting back stable cell lines

To generate rescue stable cell pools, HA-tagged FBP1 (WT, S144A or S144D) was cloned into the lentiviral pLVX-IRES-Neo vectors and co-transfected with pMD2.G and psPAX2 package vectors in HEK293T cells to produce lentiviruses, The breast cancer cells with stable FBP1 knockdown were then infected, following a selection with G418 for 2 weeks. The single stable cells were selected by reseeded into 96-well plates.

### Xenograft mouse model

The female 4 week old BALB/c nude mice were injected subcutaneously with 5×10^6^/100μL PBS FBP1 (WT, S144A or S144D) stable expression MCF-7 cells. Tumor volume was measured during the tumor growth for 3 weeks. Tumor volume was calculated according to the following formula: Tumor volume = (length×width^2^)/2. After three weeks, the mice were killed, and tumors were weighed. Finally, the tumor tissues were harvested, embedded, fixed, and prepared for H&E and IHC staining. Animal experiments were performed in strict accordance with the protocols approved by the Institutional Animal Care and Use Committee of Weifang Medical University.

### Breast cancer patient samples

The details of patient tissues samples were shown in Table S4. All experiments involving human participants were approved by the Review Board of the Affiliated Hospital of Weifang Medical University. The slides of tissues were prepared by Affiliated Hospital of Weifang Medical University.

### Statistical analysis

All statistical analyses were determined using the SPSS version 17.0 (SPSS Inc., Chicago, IL, USA). Quantitative data were presented as means ± SD. Statistical significance of Student's t-test was used for two-group comparisons. Statistical significance was displayed as *P < 0.05, and n.s. was not significant.

Other materials and methods were shown in Supplementary Data.

## Results

### PIM2 interacts with FBP1 and phosphorylates it at Ser144

**Figure 1:**
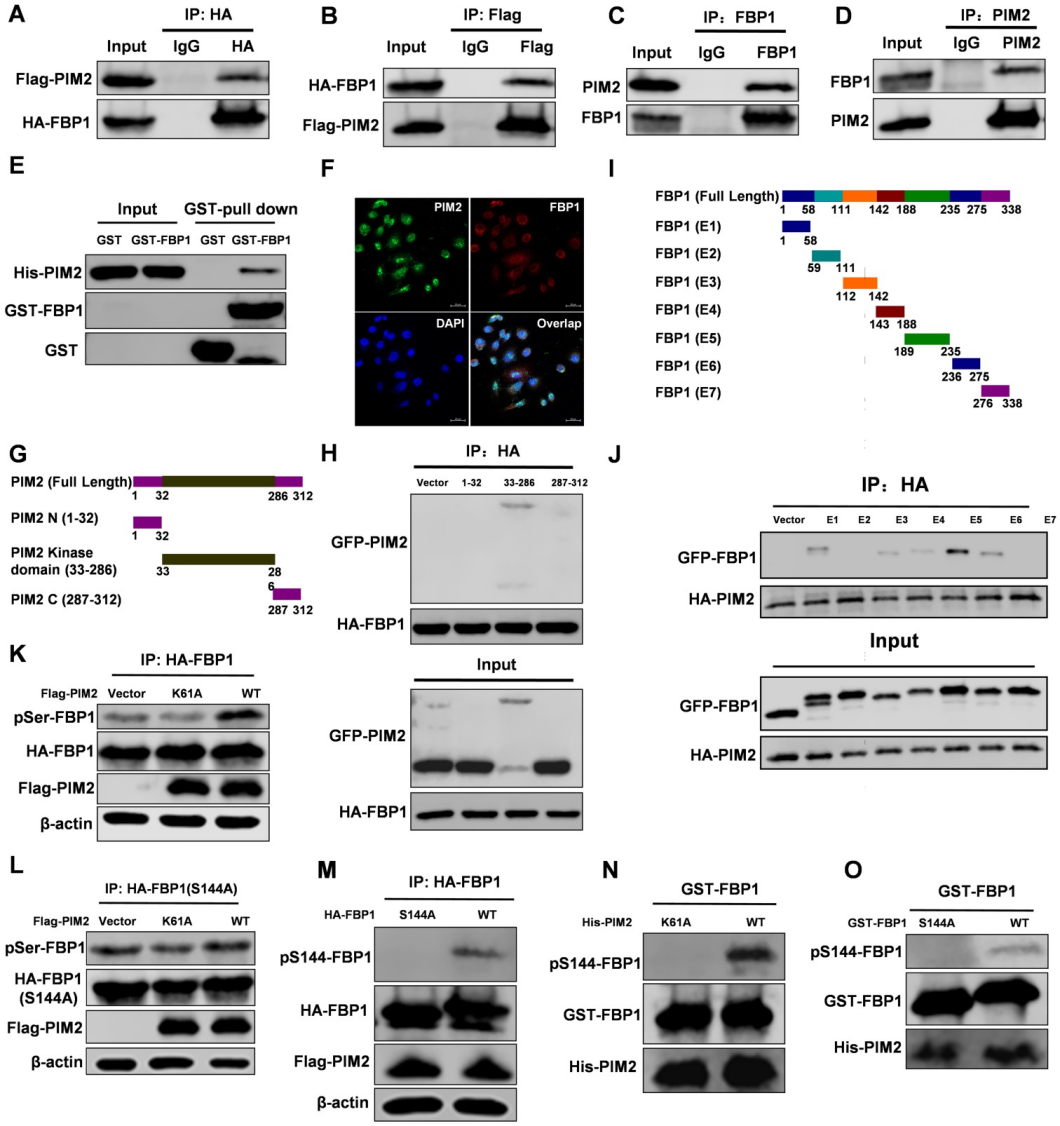
** PIM2 interacts with FBP1 and phosphorylates it at Ser144.** Immunoprecipitation and immunoblotting analyses were performed with the indicated antibodies. **(A, B)** 293T cells were transfected with indicated plasmids, followed by IP with anti-HA (a) or Flag (b) and IB with indicated antibodies. **(C, D)** MCF-7 cells were collected, followed by IP with anti-FBP1 (c) or PIM2 (d) and IB with indicated antibodies. **(E)** Purified GST-tagged FBP1 or GST was mixed with His-PIM2 for GST pull-down assay. **(F)** Confocal immunofluorescence microscopy was performed to analyze localization of PIM2 and FBP1 in MCF-7 cells. **(G)** The PIM2 truncation mutants used in this study. **(H)** 293T cells were overexpressed the indicated HA-tagged FBP1 and GFP-tagged PIM2 fragments proteins. Immunoprecipitation with an anti-HA antibody was performed. **(I)** The FBP1 truncation mutants used in this study. **(J)** 293T cells were overexpressed the indicated HA-tagged PIM2 and GFP-tagged FBP1 fragments proteins. Immunoprecipitation with an anti-HA antibody was performed. **(K)** 293T cells were overexpressed the indicated HA-tagged FBP1 and Flag-tagged PIM2 (WT or K61A) proteins. Immunoprecipitation with an anti-HA antibody was performed. **(L, M)** 293T cells were overexpressed the indicated HA-tagged FBP1 (S144A or WT) and Flag-tagged PIM2 (WT or K61A) proteins. Immunoprecipitation with an anti-HA antibody was performed. **(N, O)** Purified GST-tagged FBP1 (WT or S144A) was mixed with the indicated bacterially purified His-tagged PIM2 proteins. An *in vitro* kinase assay was performed.

We recently used PIM2 as bait to identify FBP1 as a new binding partner 15. PIM2 as an oncogene played an important role in breast tumorigenesis, but the underlying mechanism of its oncogene function remains elusive. The interaction between PIM2 and FBP1 was determined by co-immunoprecipitation (Co-IP) assay (Figure 1A-1D). In addition, a GST-pull down assay suggested that PIM2 directly interacted with FBP1 (Figure 1E). Furthermore, we used immunofluorescent staining to determine endogenous PIM2 was colocalized with endogenous FBP1 primarily in the nucleus but also slightly in the cytoplasm (Figure 1F). To determine which domains of PIM2 and FBP1 are responsible for regulating this interaction, truncated constructs of the functional domains of PIM2 and FBP1 were made for further analysis (Figure 1G and 1I) 5, 22. Co-IP analyses suggested that the kinase domain of PIM2 (33-286aa) was associated with FBP1 (Figure 1H). Moreover, PIM2 demonstrated strong binding to FBP1 domains (E1, E3, E4, E5, and E6), whereas other mutants did not bind to PIM2 (Figure 1J). Taken together, these data indicate that PIM2 physically interacts with FBP1.

As a serine/threonine kinase, PIM2 mediates tumor progression via the phosphorylation and activation of a variety of its substrate proteins 12. Thus, we evaluated whether PIM2 could phosphorylate FBP1. Wild-type PIM2 increased FBP1 serine phosphorylation levels compared with the control vector or kinase-inactive (Figure 1K) but failed to promote threonine phosphorylation levels (Figure S1A). Interestingly, we found potential PIM substrate motifs in FBP1 (Figure S1B). Moreover, phosphorylation of FBP1 at Ser144 was identified by proteomic analyses according to the protein post-translational modifications database (https://www.phosphosite.org/) 23, which was consistent with our speculation. As we expected, mutant FBP1 S144A abrogated the effect on PIM2-induced serine phosphorylation (Figure 1L). Furthermore, we produced an antibody that specifically recognizes FBP1 Ser144 phosphorylation and verified its validity via immunohistochemistry assays in breast cells and tissue samples (Figure S1C and S1D). We then used this FBP1-phosphorylation antibody and determined that PIM2 has no effect on the FBP1 S144A mutant (Figure 1M). Moreover, an *in vitro* kinase assay demonstrated that PIM2 directly phosphorylated FBP1 at Ser144 (Figure 1N and 1O). However, PIM1 and PIM3 had no effect on FBP1 Ser144 phosphorylation (Figure S1E). To further test whether this phosphorylation also happens in breast cancer cells, we used PIM inhibitor-SMI-4a. SMI-4a abrogated the effects on FBP1 Ser144 phosphorylation (Figure S1F). Taken together, our results provide convincing evidence that PIM2 is a direct kinase for FBP1.

### FBP1 Ser144 phosphorylation regulates binding to p65

**Figure 2:**
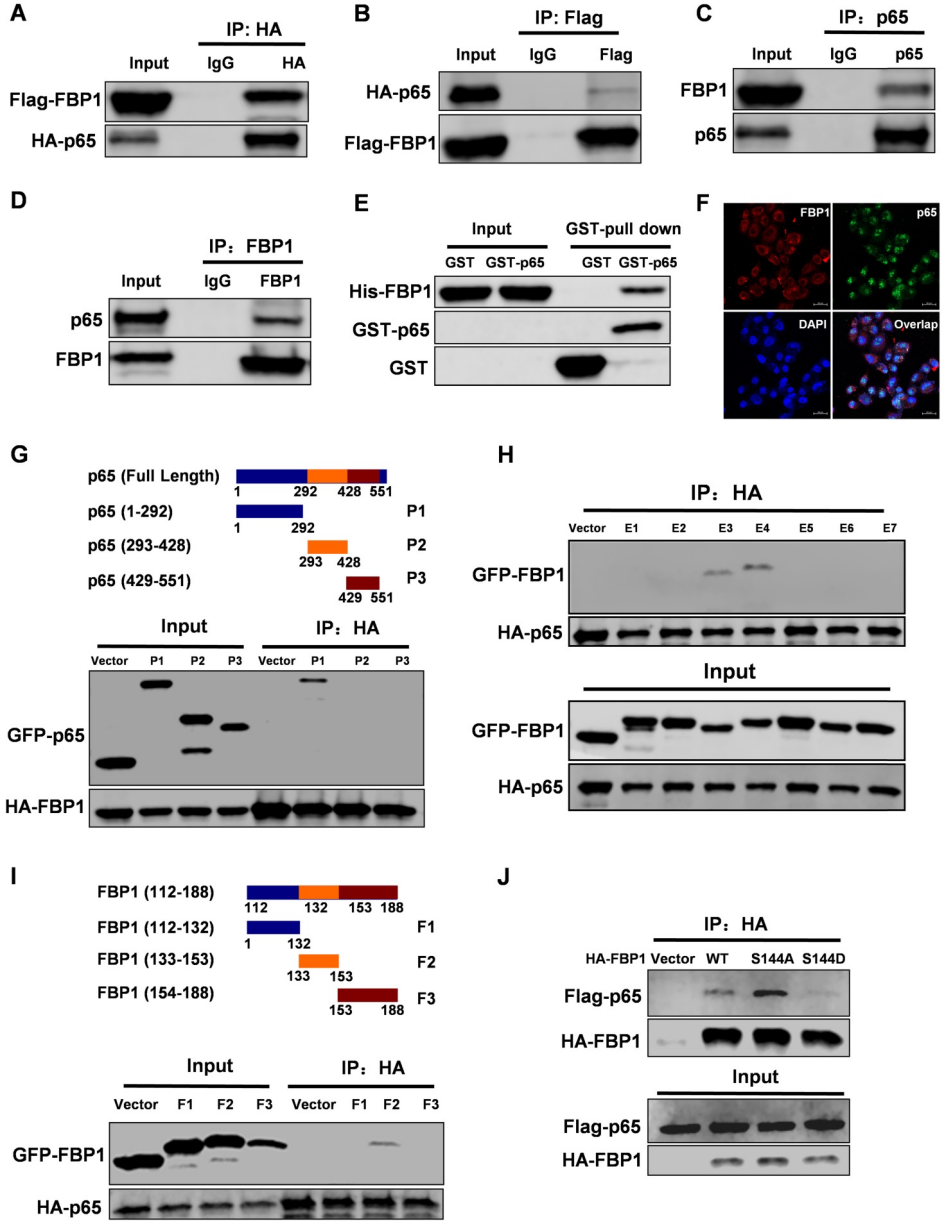
** FBP1 Ser144 phosphorylation regulates binding to p65.** Immunoprecipitation and immunoblotting analyses were performed with the indicated antibodies. **(A, B)** 293T cells were transfected with indicated plasmids, followed by IP with anti-HA (a) or Flag (b) and IB with indicated antibodies. **(C, D)** MCF-7 cells were collected, followed by IP with anti-p65 (c) or FBP1 (d) and IB with indicated antibodies. **(E)** Purified GST-tagged p65 or GST was mixed with His-FBP1 for GST pull-down assay. **(F)** Confocal immunofluorescence microscopy was performed to analyze localization of FBP1 and p65 in MCF-7 cells. **(G)** The P65 truncation mutants used in this study. 293T cells were overexpressed the indicated HA-tagged FBP1 and GFP-tagged p65 fragments proteins. Immunoprecipitation with an anti-HA antibody was performed. **(H, I)** 293T cells were overexpressed the indicated HA-tagged p65 and GFP-tagged FBP1 fragments proteins. Immunoprecipitation with an anti-HA antibody was performed. **(J)** 293T cells were overexpressed the indicated HA-tagged FBP1 (WT, S144A or S144D) and Flag-tagged p65 proteins. Immunoprecipitation with an anti-HA antibody was performed.

Interestingly, PIM2 failed to affect the enzymatic activity of FBP1 (Figure S2A). To further investigate the FBP1 non-canonical functions that are regulated by Ser144 phosphorylation, we used FBP1 as bait to screen for interaction partners. Interestingly, we found that NF-κB p65 was a new binding partner of FBP1. To further confirm their interaction, we performed a co-IP assay. The data showed that FBP1 could bind to p65 in breast cancer (Figure 2A-2D). Moreover, the enzymatic activity of FBP1 was dispensable for their interaction (Figure S2B). We next evaluated the binding with other NF-κB family proteins and found that FBP1 could interact with p50, but not RELB (Figure S2C and S2D). Furthermore, *in vitro* experiments demonstrated that FBP1 direct binding to p65 was independent of other proteins (Figure 2E). Immunofluorescence assays showed the co-localization of FBP1 and p65 in MCF-7 cells (Figure 2F). To determine which domains of FBP1 and p65 are responsible for regulating their interaction, truncated constructs of FBP1 and p65 were constructed according to their functional domains (Figure 1G and 2G). Co-IP analyses suggested that the DNA-binding domain of p65 (1-292aa) was associated with FBP1 (Figure 2G). Moreover, p65 exhibited strong binding to the domains of FBP1 (E3 and E4), whereas other mutants could not bind to p65 (Figure 2H). Furthermore, we found that the FBP1 (F2) domain containing the PIM2 phosphorylation site interacted with p65 (Figure 2I). Next, we tested whether this phosphorylation contributed to their interaction. As we expected, phosphorylation of the PIM2 site abrogated FBP1 interaction with p65 (Figure 2J). Thus, these data demonstrate that PIM2 phosphorylation of FBP1 inhibits its interaction with p65.

### FBP1 Ser144 phosphorylation regulates p65 stability via the CHIP-mediated ubiquitin proteasome pathway

**Figure 3:**
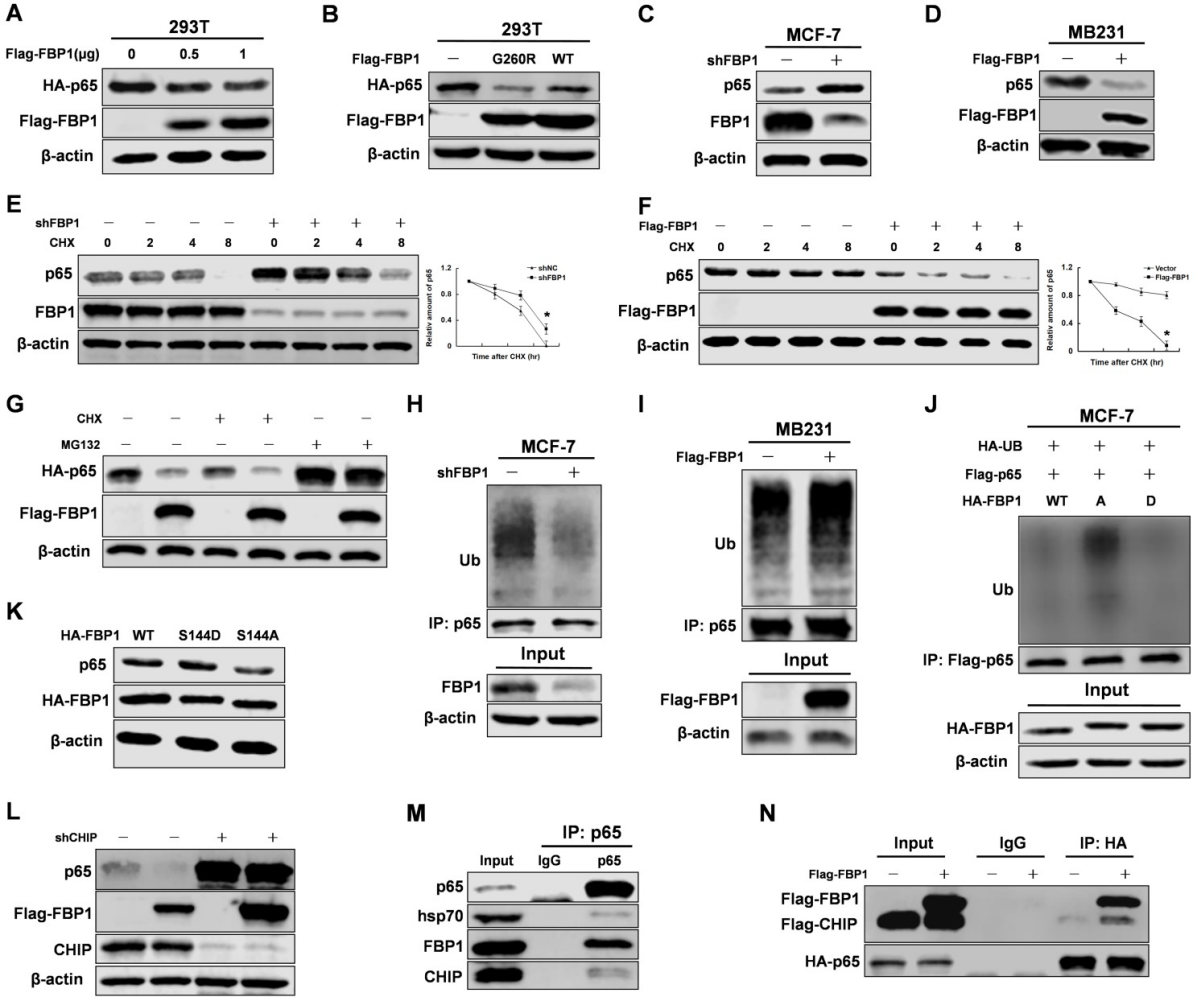
** FBP1 Ser144 phosphorylation regulates p65 stability via the CHIP-mediated ubiquitin proteasome pathway.** Immunoprecipitation and immunoblotting analyses were performed with the indicated antibodies. **(A, B)** 293T cells were overexpressed the indicated HA-tagged p65 and Flag-tagged FBP1 (WT or G260R) proteins. Total cell lysates were prepared. **(C)** MCF-7 cells were knocked down FBP1 with shRNA. Total cell lysates were prepared. **(D)** MB231 cells were overexpressed the indicated Flag-tagged FBP1 proteins. Total cell lysates were prepared. **(E)** MCF-7 cells with stable knockdown FBP1 proteins were treated with CHX for indicated time. Total cell lysates were prepared. (All data represent mean ± SEM n = 3), *p<0.05. **(F)** MB231 cells with overexpressed Flag-tagged FBP1 proteins were treated with CHX for indicated time. Total cell lysates were prepared. (All data represent mean ± SEM n = 3), *p<0.05. **(G)** 293T cells with overexpression of the indicated both Flag-tagged FBP1 and HA-tagged p65 proteins were treated with CHX or CHX+MG132 for 12hr. Total cell lysates were prepared. **(H)** MCF-7 cells were knocked down FBP1 by shRNA. Total cell lysates were prepared. Immunoprecipitation with an anti-p65 antibody was performed. **(I)** MB-231 cells were over-expressed Flag-tagged FBP1. Total cell lysates were prepared. Immunoprecipitation with an anti-p65 antibody was performed. **(J)** MCF-7 cells were co-overexpressed the indicated Flag-tagged p65 and HA-tagged FBP1 (WT, S144A or S144D) proteins. Total cell lysates were prepared. Immunoprecipitation with an anti-Flag antibody was performed. **(K)** MCF-7 cells were overexpressed HA-tagged FBP1 (WT, S144A or S144D) proteins. Total cell lysates were prepared. **(L)** MCF-7 cells with CHIP knocked down were overexpressed the indicated Flag-tagged FBP1 proteins. Total cell lysates were prepared. **(M)** MCF-7 cell lysates were prepared. **(N)** MCF-7 cells with overexpression of the indicated both Flag-tagged PIM2 or CHIP and HA-tagged p65 proteins. Total cell lysates were prepared.

Previous studies found that FBP1 regulated the protein stability of some transcriptional factors 10, 11. To determine whether FBP1 regulates p65 protein stability, we examined the effect of FBP1 manipulation on p65 protein levels. We found that FBP1 decreases p65 protein levels in a dose-dependent manner (Figure 3A). Moreover, mutant FBP1 G260R had an inhibitory effect on p65 protein levels similar to that of wild-type FBP1, suggesting that its enzymatic activity was not required for this regulation (Figure 3B). Because our previous study demonstrated that FBP1 protein expression was higher in MCF-7 cells than in MB231 cells, MCF-7 cells were used for knockdown, and MB231 cells were used for overexpression (Figure S3A) 11. *FBP1* knockdown efficiency was validated in MCF-7 cells (Figure S3B), and the data showed that FBP1 negatively mediated endogenous p65 protein levels in breast cancer cells (Figure 3C and 3D). To further determine whether FBP1 regulates p65 stability, we next examined the protein half-life of p65 in response to the manipulation of FBP1 levels in the presence of cycloheximide to inhibit new protein synthesis. *FBP1* knockdown significantly increased the protein half-live of p65 (Figure 3E), whereas the opposite effect on its half-life was seen with FBP1 overexpression (Figure 3F).

We next investigated whether the proteasome pathway was involved in regulating of p65 protein stability. Our data suggested that MG132 blocked the FBP1-mediated degradation of p65 protein (Figure 3G). The proteasome pathway often increases protein ubiquitination levels, so we tested whether FBP1 regulated p65 ubiquitination levels. Consistent with previous results, FBP1 significantly enhanced p65 ubiquitination (Figure 3H and 3I). To evaluate if FBP1 Ser144 phosphorylation is required for regulation p65 protein stability, we over-expressed HA-tagged FBP1 (WT, S144A or S144D) in MCF-7 cells. Consistently, FBP1 Ser144 phosphorylation increased p65 protein levels via reducing p65 ubiquitination (Figure 3J and 3K). According to previous studies, CHIP binds to p65 and promotes its ubiquitination and degradation through the proteasome pathway 24, 25. To investigate whether CHIP is responsible for FBP1-mediated p65 degradation, we first determined whether this degradation was affected by CHIP. Indeed, the FBP1-mediated p65 degradation was abrogated by CHIP knockdown (Figure 3L). Finally, p65 interacted with the CHIP cooperative protein-Hsp70 (Figure 3M), and FBP1 overexpression enhanced CHIP interaction with p65 (Figure 3N). Again, FBP1 Ser144 phosphorylation reduced its affinity with CHIP (Figure S3C). However, FBP1 Ser144 phosphorylation enhanced p65 protein stability, and PIM2 had no effect on FBP1 protein level (Figure S3D). Taken together, these data demonstrate that FBP1 Ser144 phosphorylation regulates p65 stability via the CHIP-mediated ubiquitin proteasome pathway.

### FBP1 Ser144 phosphorylation contributes to p65 transcriptional activity

**Figure 4:**
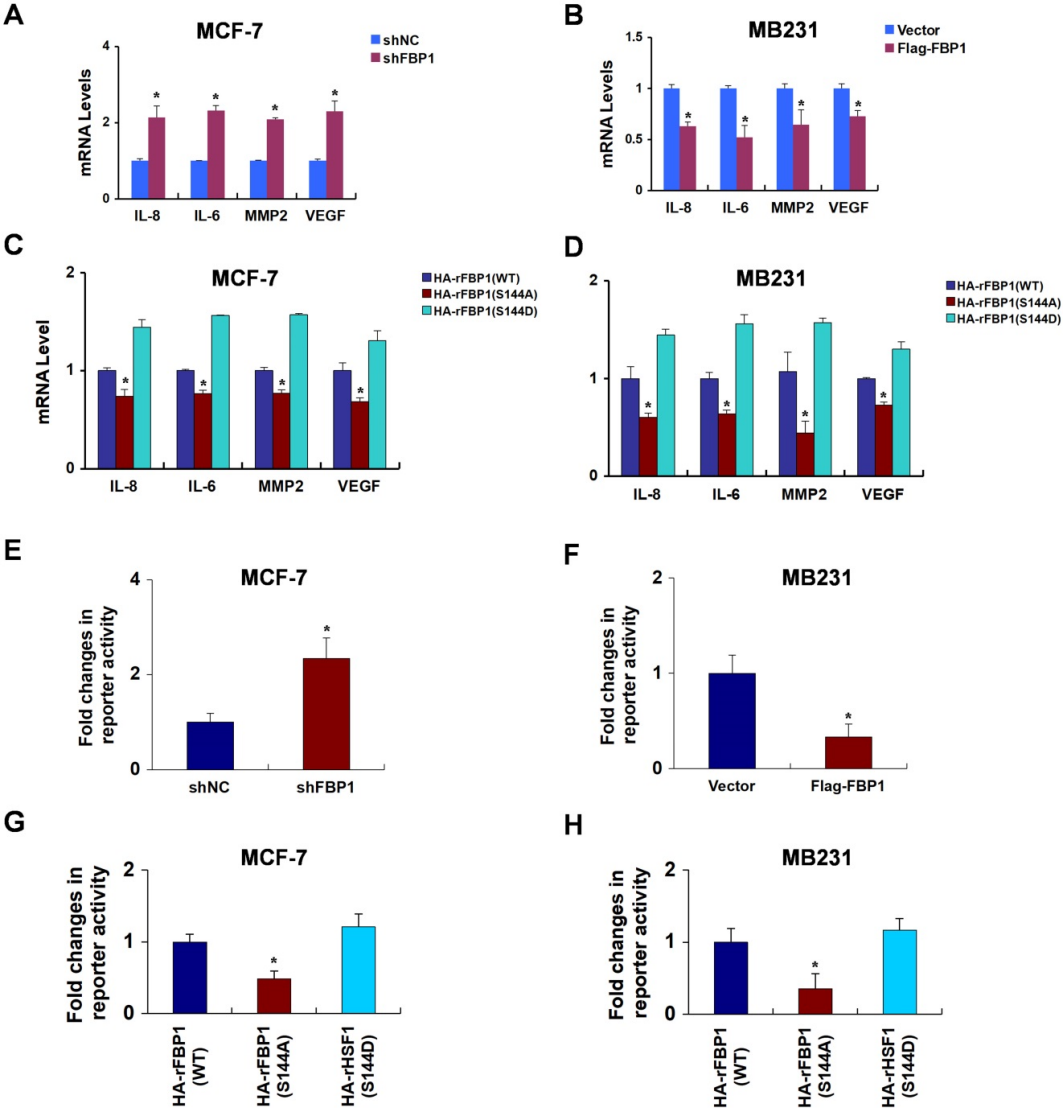
** FBP1 Ser144 phosphorylation contributes to p65 transcriptional activity. (A)** MCF-7 cells were stably knocked down FBP1 by shRNA. mRNA levels were quantitated by RT-PCR. **(B)** MB-231 cells were stably over-expressed HA-tagged FBP1. mRNA levels were quantitated by RT-PCR. **(C, D)** MCF-7 or MB231 cells expressing FBP1 shRNA were reconstituted expression of HA-rFBP1 (WT, S144A or S144D). mRNA levels were quantitated by RT-PCR. **(E)** Stable knockdown FBP1 MCF-7 cells were transfected with dual p65 reporter plasmids, and detected by luciferase reporter assay. **(F)** Stable overexpression HA-tagged FBP1 MB231 cells were transfected with dual p65 reporter plasmids, and detected by luciferase reporter assay. **(G)** FBP1-depleted MCF-7 cells with reconstituted expression of HA-FBP1 (WT, S144A or S144D) were transfected with dual p65 reporter plasmids, and detected by luciferase reporter assay. **(H)** FBP1-depleted MB231 cells with reconstituted expression of HA-FBP1 (WT, S144A or S144D) were transfected with dual p65 reporter plasmids, and detected by luciferase reporter assay. (All data represent mean ± SEM n = 3), *p<0.05.

Our study demonstrated that FBP1 binds to the DNA binding domain of p65. Thus, we predicted that the manipulation of FBP1 would affect p65 transcriptional progression. To examine this notion, we rescued FBP1 expression in FBP1-knockdown breast cancer cells (Figure S4A and S4B). Previously reported p65 transcriptional target genes, such as *IL-8*, *IL-6*, *MMP2*, and *VEGF*, emerged as responsive sensitive to FBP1 manipulation (Figure 4A and 4B). Compare with the mutant FBP1 S144A, wide type FBP1 and mimic FBP1 S144D enhanced the expression of p65 transcriptional target genes, suggesting FBP1 phosphorylation by PIM2 contributed to p65 transcriptional activity (Figure 4C and 4D). Moreover, luciferase reporter assay confirmed that FBP1 knockdown or overexpression affected p65 transcriptional activity (Figure 4E and 4F). Similarly, p65 transactivation activity increased upon FBP1 Ser144 phosphorylation (Figure 4G and 4H). Taken together, we conclude that FBP1 Ser144 phosphorylation leads to enhanced p65 transcriptional activity in breast cancer cells.

### FBP1 Ser144 phosphorylation promotes p65-induced PD-L1 expression

**Figure 5:**
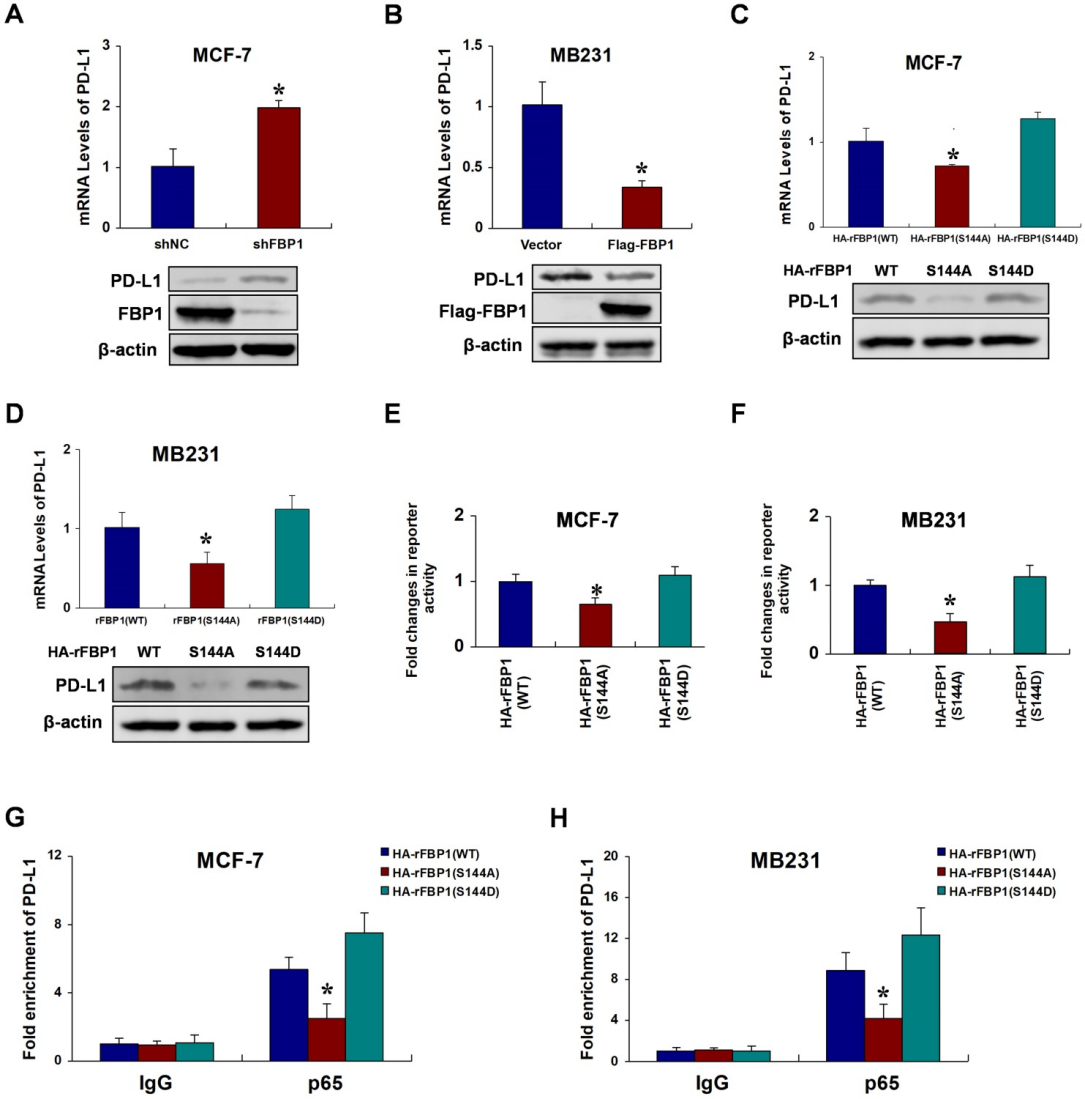
** FBP1 Ser144 phosphorylation promotes p65-induced PD-L1 expression. (A)** MCF-7 cells were stably transfected with FBP1 shRNA. PD-L1 mRNA levels were quantitated by RT-PCR. **(B)** MB231 cells were transfected with Flag-tagged FBP1. PD-L1 mRNA levels were quantitated by RT-PCR. **(C, D)** FBP1-depleted MCF-7 or MB231 cells were reconstituted expression of HA-rFBP1 (WT, S144A or S144D). PD-L1 mRNA levels were quantitated by RT-PCR. **(E, F)** Reconstituted expression of HA-rFBP1 (WT, S144A or S144D) MCF-7 or MB231 cells were transfected with dual PD-L1 reporter plasmids, and detected by luciferase reporter assay. **(G, H)** CHIP assays were performed using FBP1-depleted MCF-7 or MB231 cells with reconstituted expression of HA-rFBP1 (WT, S144A or S144D). The results were normalized against the values of IgG controls. (All data represent mean ± SEM n = 3), *p<0.05.

Previous studies have identified programmed death ligand 1 (PD-L1) as a target gene of p65 26, 27. Thus, we speculated that FBP1 Ser144 phosphorylation regulates PD-L1 expression via p65. To investigate the potential relationships between FBP1 and PD-L1, we first silenced or overexpressed FBP1 to test PD-L1 expression in breast cancer cells. The results showed that FBP1 repressed PD-L1 expression (Figure 5A and 5B). We further examined whether PD-L1 expression was regulated by FBP1 Ser144 phosphorylation. Compare with that of the mutant FBP1 S144A, the ectopic expression of wild type FBP1 substantially increased PD-L1 expression, and this effect was largely enhanced in cells expressing the mutant FBP1 S144D (Figure 5C and 5D). Consistently, luciferase reporter assays demonstrated that FBP1 Ser144 phosphorylation highlights p65 transactivation activity (Figure 5E and 5F). Moreover, we performed chromatin immunoprecipitation assays, and the results suggested that FBP1 Ser144 phosphorylation enhanced p65 binding to the PD-L1 promoter (Figure 5G and 5H). To further validate whether FBP1 Ser144 phosphorylation regulates PD-L1 expression depending on p65, we knocked out p65 using a special single guide RNA. We found that p65 was involved in FBP1 Ser144 phosphorylation regulating PD-L1 expression (Figure S5A and S5B). These data indicate that FBP1 Ser144 phosphorylation augments p65-induced PD-L1 expression.

### FBP1 Ser144 phosphorylation promotes breast tumorigenesis

**Figure 6:**
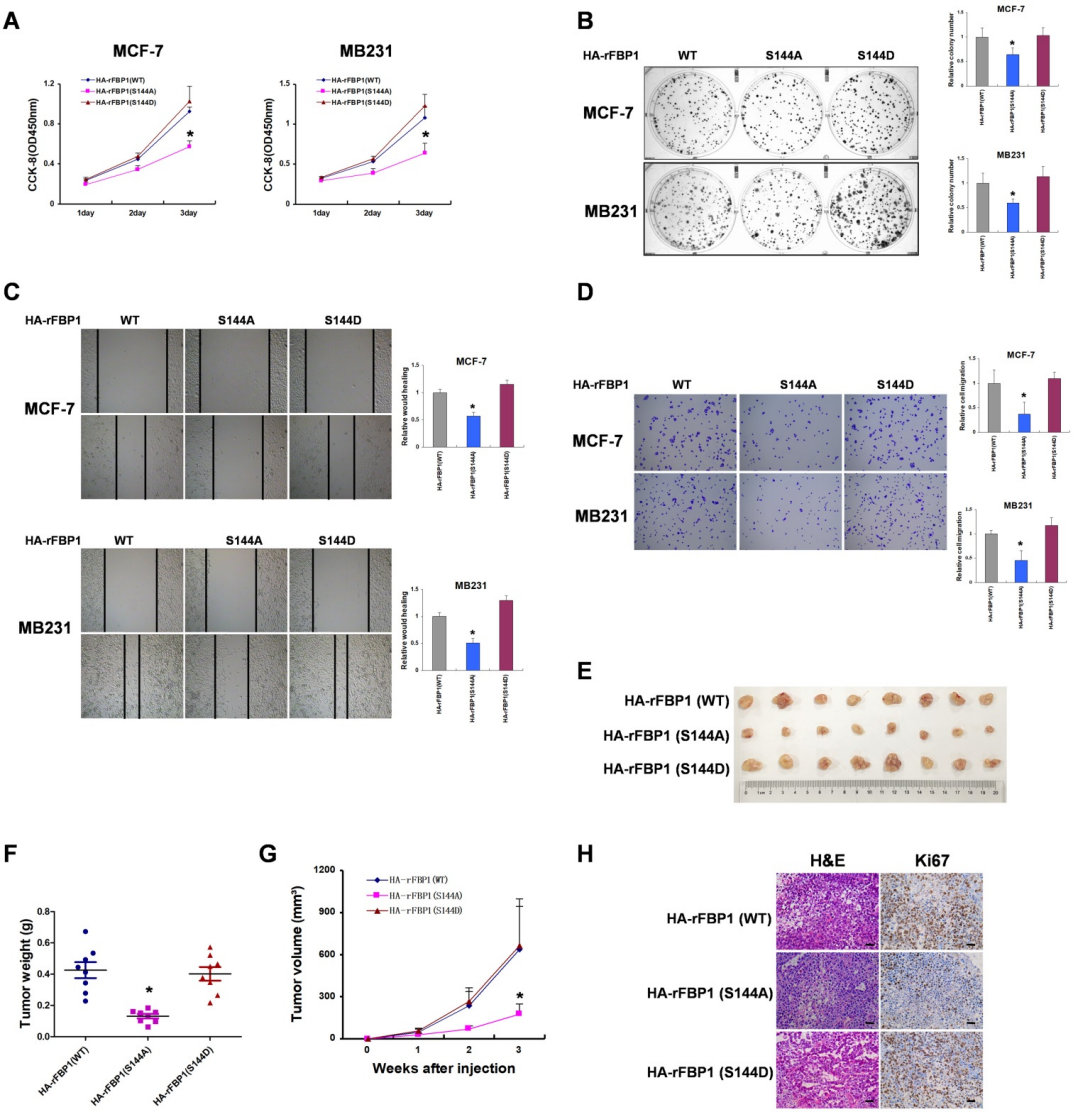
** FBP1 Ser144 phosphorylation promotes breast tumorigenesis. (A)** MCF-7 or MB231 cells with stable expression of HA-rFBP1 (WT, S144A or S144D) were seeded in a new plate. CCK-8 assay was performed to determine cell proliferation. **(B-D)** MCF-7 or MB231 cells with stable expression of HA-rFBP1 (WT, S144A or S144D) were seeded in a new plate. Clone formation, wound healing assay and cell invasion assays were performed. **(E-G)** MCF-7 cells with stable expression of HA-rFBP1 (WT, S144A or S144D) were subcutaneously injected into nude mice. After 3 weeks, the mice were sacrificed and dissected at the endpoint. Tumor growth and weight were examined. **(H)** Representative images of H/E staining and Ki67 staining of tumor samples (Scale bar, 20μm). (All data represent mean ± SEM n = 3), *p<0.05.

To investigate the biological significance of FBP1 Ser144 phosphorylation, we measured the effect of FBP1 Ser144 phosphorylation on breast tumorigenesis *in vitro*. These results showed that FBP1 Ser144 phosphorylation promoted cell proliferation in breast cancer cells (Figure 6A and 6B). Consistently, FBP1 Ser144 phosphorylation increased cell migration and invasion (Figure 6C and 6D). Furthermore, FBP1 Ser144 phosphorylation stimulated breast tumor growth *in vivo*, as detected in athymic nude mice (Figure 6E-6G). Again, we used immunohistochemical analysis to demonstrate low Ki67 expression in mutant FBP1 S144A, which could reflect the proliferative ability of cells. Collectively, these data suggest that FBP1 Ser144 phosphorylation contributes to breast tumorigenesis.

### FBP1 Ser144 phosphorylation is upregulated in breast cancer

**Figure 7:**
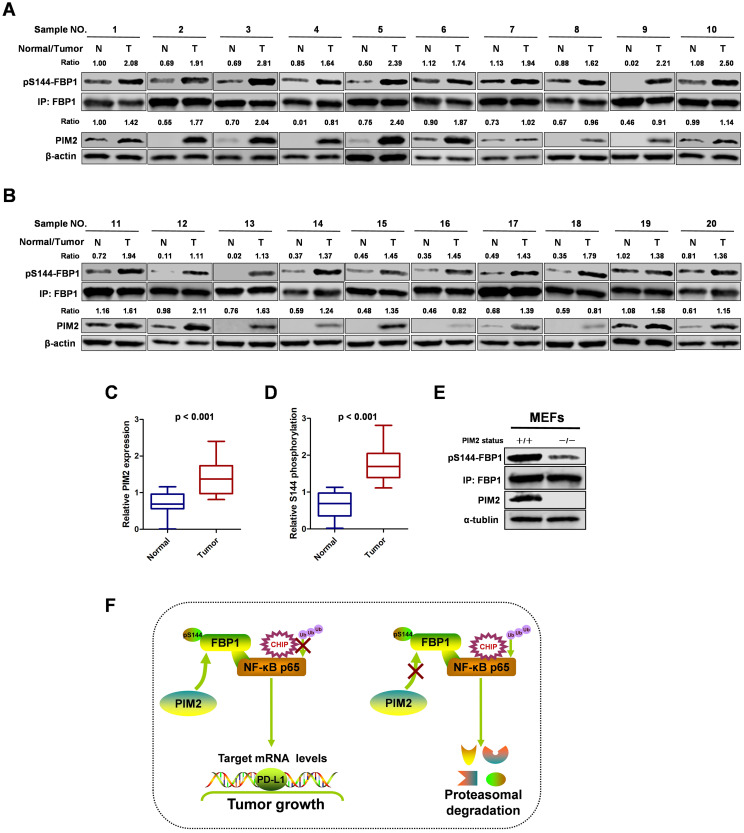
** FBP1 Ser144 phosphorylation is upregulated in breast cancer. (A, B)** The expression of PIM2 and FBP1 Ser144 phosphorylation in 20 breast cancer samples (T) with paired adjacent normal tissues (N) was analyzed by IB. PIM2 was quantified and normalized to β-actin. Ser144 phosphorylation of immunopurified FBP1 was determined and normalized to FBP1 protein (Ratio). **(C, D)** PIM2 and FBP1 Ser144 phosphorylation protein levels in normal and tumor tissues were statistically analyzed. **(E)** PIM2 depletion causes decrease of FBP1 Ser144 phosphorylation protein level. Primary MEFs generated from E12.5-13.5 embryos with PIM2 knockout were followed by IB with the indicated antibodies. **(F)** Working model for PIM2-induced phosphorylation of FBP1. (All data represent mean ± SEM n = 3), *p<0.05.

To study the clinical relevance of FBP1 Ser144 phosphorylation, we collected 20 breast cancer samples with paired surrounding normal breast tissues. We analyzed PIM2 and FBP1 Ser144 phosphorylation expression levels by western blot in breast tumors (T) and their adjacent normal tissues (N) (Figure 7A and 7B). Consistently, PIM2 and FBP1 Ser144 phosphorylation were expressed at higher levels in the tumor samples than in the normal control samples (Figure 7C and 7D). Finally, we generated a *PIM2* knockout mouse model and used mouse embryonic fibroblasts (MEFs) derived from the embryos for further analysis. Indeed, PIM2 depletion caused a reduction in FBP1 Ser144 phosphorylation levels (Figure 7E). These data further support the crucial role of FBP1 Ser144 phosphorylation in breast cancer development.

## Discussion

PIM2, a serine/threonine kinase, has been shown to be highly expressed in many cancers, including breast cancer 22, liver cancer 28, stomach cancer 29, lymph cancer 30, ovarian cancer 31, endometrial cancer 16, prostate cancer 32, and lung cancer 33. In addition, PIM2 plays an important role in tumor progression by phosphorylating its downstream substrate proteins. Our data demonstrate that PIM2 interacts with FBP1 and phosphorylates it at Ser144, inhibiting FBP1 binding to p65 and enhancing its protein stability. Moreover, the effects of FBP1 Ser144 phosphorylation on downstream signaling cascades result in phenotypic changes related to breast tumorigenesis and progression. Lastly, breast cancer tissues exhibited higher PIM2 and FBP1 Ser144 phosphorylation expression than normal, tumor-adjacent breast tissues, suggesting its importance for breast cancer progression.

FBP1 functions as a tumor suppressor via the promotion of glycogen synthesis and inhibition of glycolysis in many types of cancer 34. However, the non-canonical functions of FBP1 are independent of its enzymatic activity. For example, noncanonical FBP1 acts as a co-suppressor for many transcriptional factors, including HIF-1α 5, β-catenin 35, NOTCH1 11, STAT3 36 and c-MYC 10, and it also regulates their functions through direct interactions, possibly leading to protein degradation. In addition, FBP1 binds to the WW domain of IQGAP1, and impedes IQGAP1-dependent ERK1/2 phosphorylation independent of FBP1 enzymatic activity 37. However, the posttranscriptional modification of noncanonical FBP1 has never been elucidated in breast cancer. Our findings indicate that PIM2 phosphorylates FBP1 at the Ser144 site to perform its non-canonical function of regulating p65 and PD-L1 expression.

A recent study revealed a new tumor suppressor function of FBP1 to inhibit PD-L1 expression and enhance cancer immunity 36. They found that FBP1 inhibits STAT3-dependent PD-L1 transcription, a finding that is consistent with our discovery in breast cancer. Thus, we do not rule out that other regulatory pathways are involved in FBP1-mediated PD-L1 expression. Moreover, phosphorylation is one of the most important post-translational modifications for tumor progression 38. However, to the best of our knowledge, the phosphorylation of FBP1 has yet to be reported in cancer. Therefore, this study is the first to report PIM2 phosphorylates FBP1 at Ser144, uncovering a new pathway to regulate the non-canonical functions of FBP1.

NF-κB p65 is a highly expressed transcription factor in cancer 39-41. NF-κB p65 is activated by many cytokines, including IL-8, IL-6, TNFα and many others 42, 43. Besides regulating some canonical pro-growth genes, NF-κB p65 is also known to mediate *PD-L1* mRNA level in multiple types of cancers 26, 44. However, although PIM2 can directly phosphorylate p65 and enhance its transcriptional activity, there is no indirect pathway to regulate p65. In the present study, we identified an indirect pathway wherein phosphorylated FBP1 acts as a major promoter of p65 activity and *PD-L1* transcription.

In summary, our results demonstrate that the phosphorylation of non-canonical FBP1 by PIM2 promotes breast tumorigenesis via augmenting NF-κB transcriptional activity and PD-L1 expression (Figure 7F). Our findings further suggest that PIM2-mediated non-canonical FBP1 phosphorylation may be targeted in breast cancer therapies.

## Supplementary Material

Supplementary figures, tables, methods.

## Abbreviations

Co-IP: co-immunoprecipitation
FBP1: fructose-1,6-bisphosphatase 1
HIF-1α: hypoxia inducible factor 1α
PD-L1: programmed death ligand 1
