# Supplementary material for: Fructose-1, 6-bisphosphatase 1 interacts with NF-κB p65 to regulate breast tumorigenesis via PIM2 induced phosphorylation
Source: Theranostics. 2020 Jul 9;10(19):8606–18. doi: 10.7150/thno.46861 (PMC7392005; doi:10.7150/thno.46861)
Supplement: Supplementary file 1 — Supplementary figures, tables, methods. [file thnov10p8606s1.pdf]

Supplementary Data

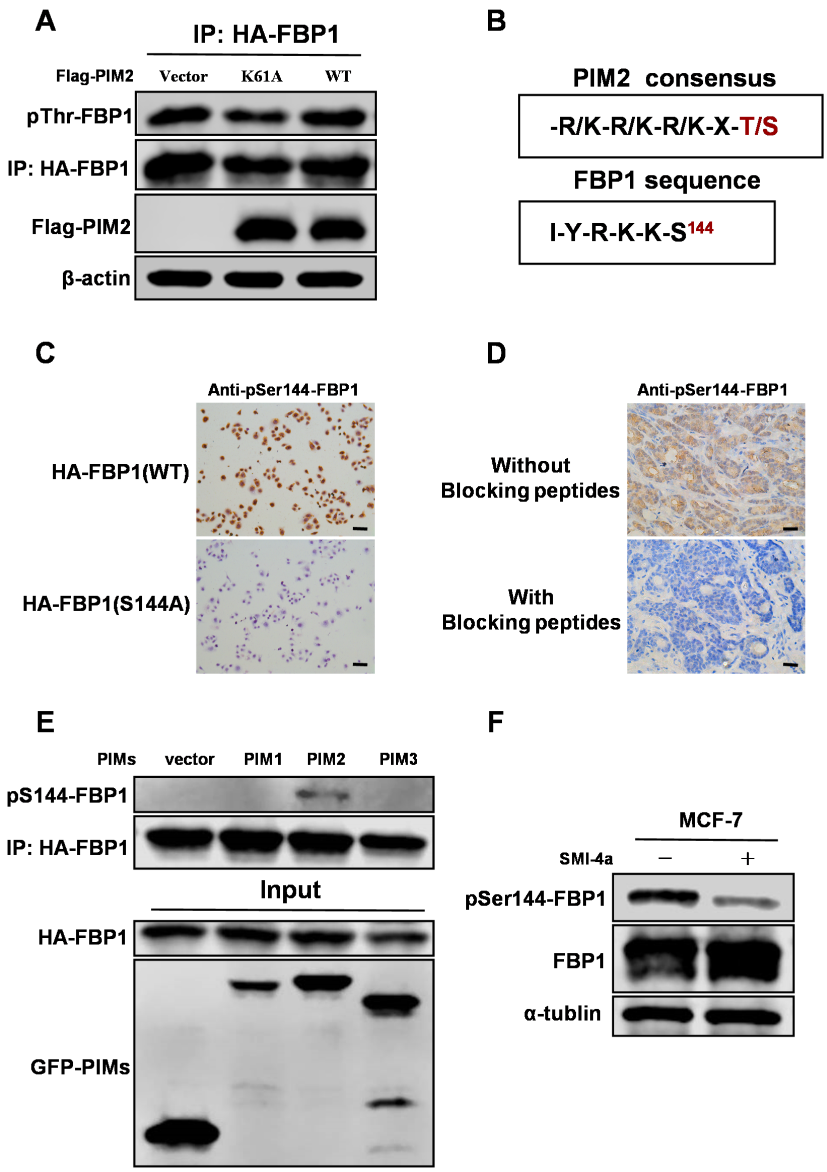

Supplementary Figure 1. (A) HEK293T cells were co-transfected with HA-tagged FBP1 and Flag-tagged PIM2 (WT or K61A) proteins. Immunoprecipitation with an anti-HA antibody was performed followed by western blot with indicated antibodies. **(B)** A putative PIM2 substrate motif was identified in FBP1. **(C)** Validation of the anti-pS144-FBP1 antibody by immunohistochemistry analyses of MCF-7 cells with transfections of HA-tagged FBP1 (WT or S144A) (scale bar, 20 μm). **(D)** Validation of the anti-pS144-FBP1 antibody by immunohistochemistry analyses of human breast cancer tissues，which were performed with the indicated antibody with or without blocking peptides against FBP1 S144 (scale bar, 20 μm). **(E)** HEK293T cells were co-transfected HA-tagged FBP1 with GFP-tagged PIM1, PIM2, or PIM3. Immunoprecipitations with an anti-HA antibody were performed, and immunoblotting analyses were performed with the indicated antibodies. **(F)** MCF-7 cells were treated with SMI-4a (10μM) for 24hr. The immunoblotting analyses were performed with the indicated antibodies.

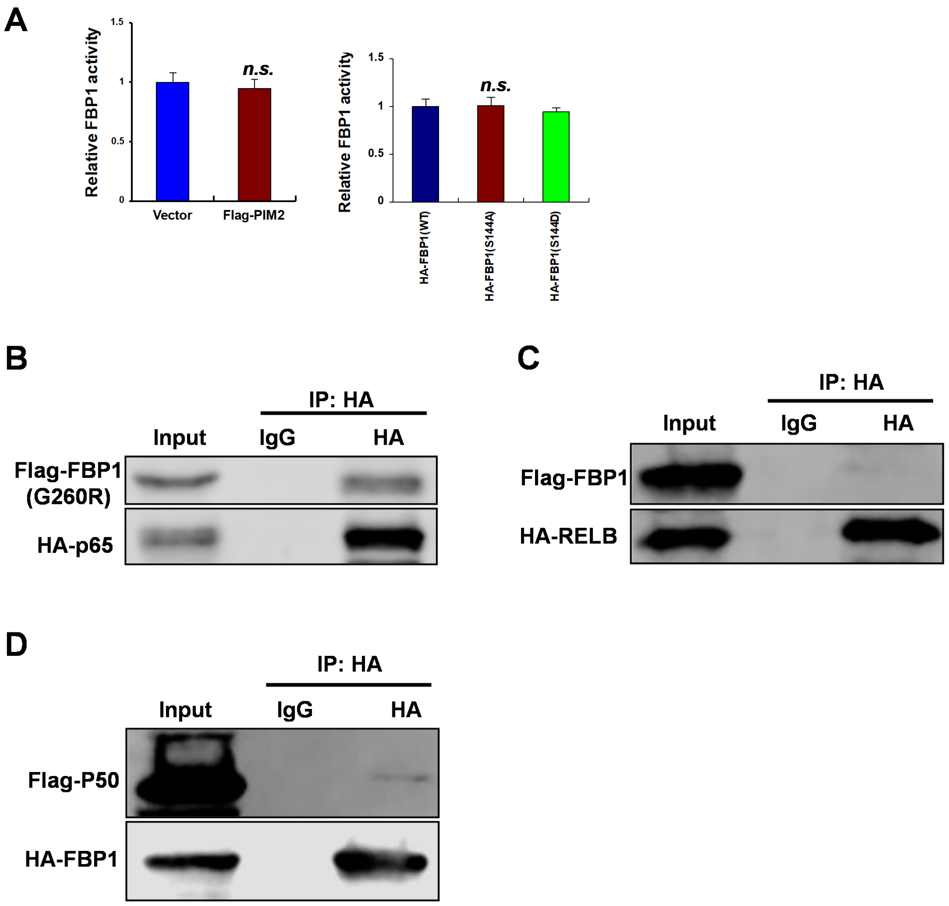

Supplementary Figure 2. (A) 293T cells were co-transfected with HA-tagged FBP1 and Flag-tagged PIM2, or HA-FBP1 (WT, S144A or S144D). FBP1 enzymatic activity in the lysates was detected. **(B)** 293T cells were transfected with indicated plasmids, followed by IP with anti-HA and IB with indicated antibodies. **(C)** 293T cells were transfected with indicated plasmids, followed by IP with anti-HA and IB with indicated antibodies. **(D)** 293T cells were transfected with indicated plasmids, followed by IP with anti-HA and IB with indicated antibodies.

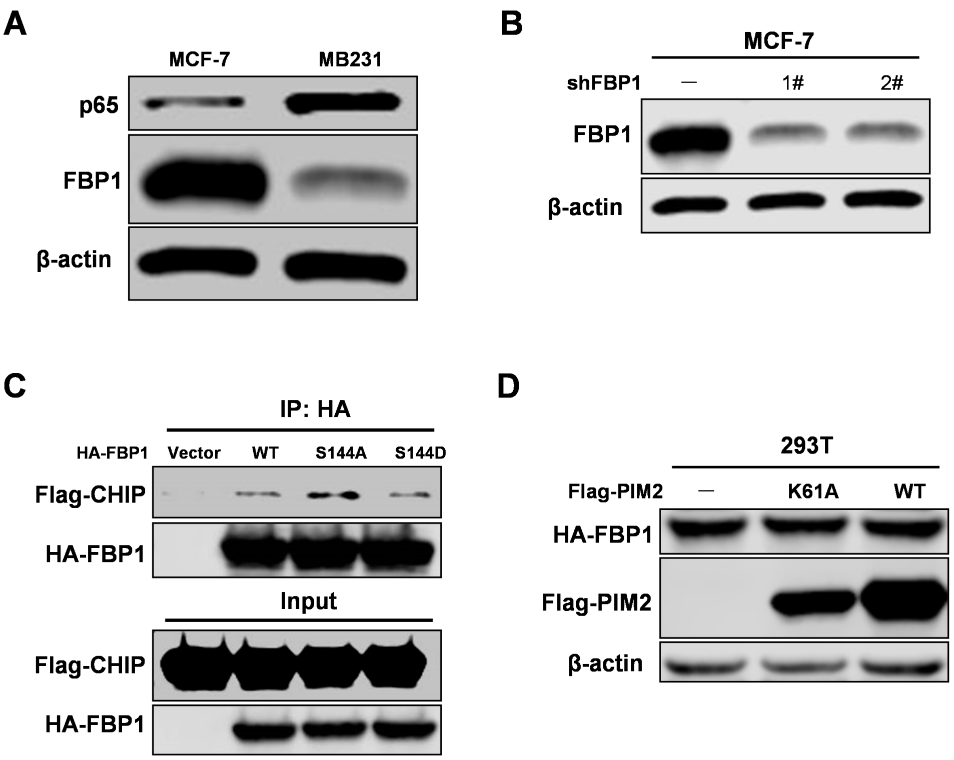

Supplementary Figure 3. **(A)** MCF-7 or MB231 cells were collected followed by western blot. **(B)** FBP1 was stably knocked down in MCF-7 cells followed by western blot. **(C)** IB analysis of Inputs and IPs derived from 293T cells transfected with indicated plasmids. **(D)** 293T cells were transfected with indicated plasmids, followed by IB.

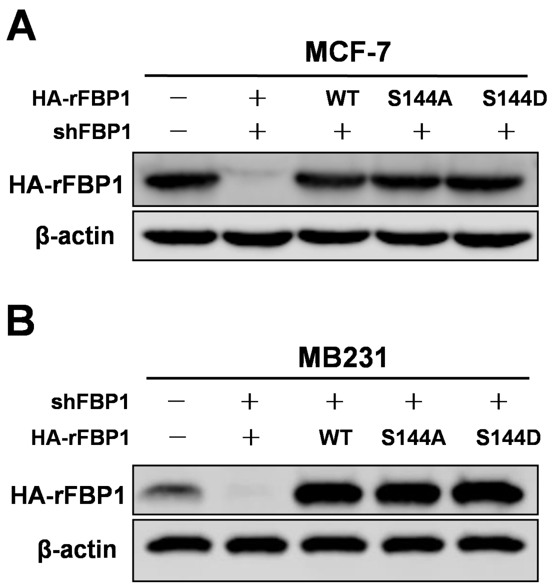

Supplementary Figure 4. (A) FBP1-depleted MCF-7 cells were reconstituted with the indicated protein expression. Immunoblotting analyses were performed with the indicated antibodies. **(B)** FBP1-depleted MB231 cells were reconstituted with the indicated protein expression. Immunoblotting analyses were performed with the indicated antibodies.

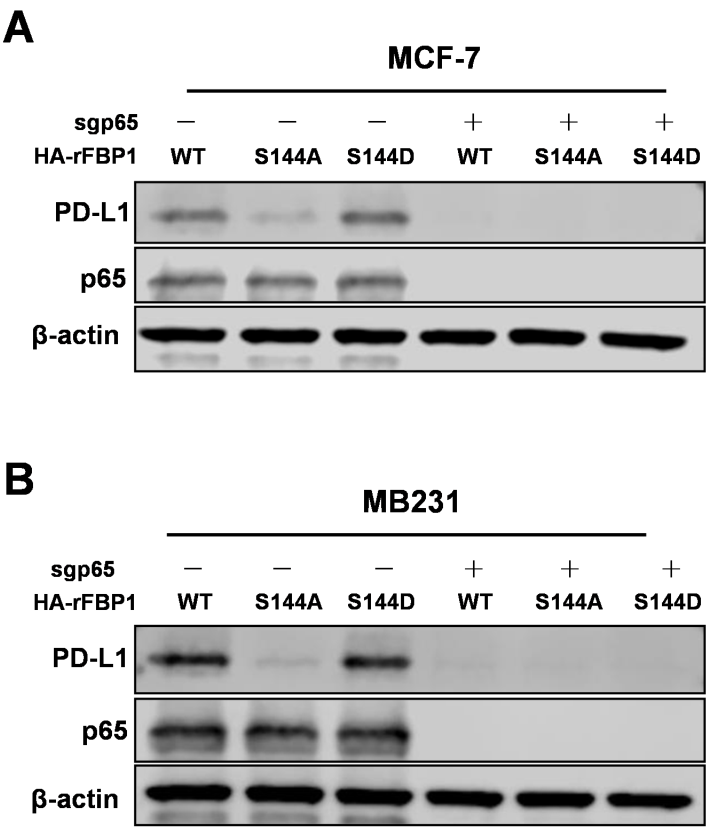

Supplementary Figure 5. (A) Rescued indicated FBP1 MCF-7 cells were infected with sgRNA-GFP control or sgRNA-p65 lentiviral. The infected cells were selected with 1 μg/mL puromycin for 72hr to eliminate the non-infected cells before harvesting. **(B)** Rescued indicated FBP1 MB231 cells were infected with sgRNA-GFP control or sgRNA-p65 lentiviral. The infected cells were selected with 1 μg/mL puromycin for 72hr to eliminate the non-infected cells before harvesting.

Table S1. Primary antibodies and reagents used in this study.

REAGENT or RESOURCE	SOURCE	IDENTIFIER
**Antibodies**		
Rabbit anti-pThr144 FBP1	Dia-An, Inc, in Wuhan, in China	N/A
Mouse anti-HA	Sigma-Aldrich	Cat#H3663
Mouse anti-Flag	Sigma-Aldrich	Cat#F1804
Mouse anti-GFP	Sigma-Aldrich	Cat#G6539
Mouse anti-β-actin	Sigma-Aldrich	Cat#A1978
Rabbit anti-HA	Proteintech	Cat#51064-2-AP
Rabbit anti-Flag	Proteintech	Cat#20543-1-AP
Rabbit anti-GFP	Proteintech	Cat#50430-2-AP
Rabbit anti-β-actin	Proteintech	Cat#20536-1-AP
Mouse anti-PD-L1	Proteintech	Cat#66248-1-Ig
Mouse anti-FBP1	Sigma-Aldrich	Cat#SAB1405798
Mouse anti-PIM2	Santa Cruz	Cat#sc-13514
Mouse anti-p65	Santa Cruz	Cat#sc-8008
Rabbit anti-FBP1	Sigma-Aldrich	Cat#SAB1410372
Rabbit anti-PIM2	Abcam	Cat#ab129193
Rabbit anti-p65	Proteintech	Cat#10745-1-AP
Mouse anti-GST	Proteintech	Cat#66001-2-Ig
Mouse anti-HIS	Proteintech	Cat#66005-1-Ig
Rabbit anti-hsp70	Proteintech	Cat#10995-1-AP
Rabbit anti-Ubiquitin	Abcam	Cat#ab134953
Rabbit anti-CHIP	Proteintech	Cat#55430-1-AP
Rabbit anti-phosphoserine	Abcam	Cat#ab9332
Mouse anti-phosphothreonine	Cell signaling	Cat#9386
IRDye 800CW goat anti-rabbit	LI-COR	Cat#925-32210
IRDye 680LT goat anti-mouse	LI-COR	Cat#925-68020
Rabbit anti-Ki67	Abcam	Cat#ab92742
ANTI-FLAG Affinity Gel	Sigma-Aldrich	Cat#F2426
Anti-HA Affinity Gel	Sigma-Aldrich	Cat#E6779
Pierce™ Glutathione Agarose	Thermo Fisher	Cat#16100
Ni-NTA Agarose	Thermo Fisher	Cat#R90101
MagnaBind™ Protein A Beads	Thermo Fisher	Cat#21348
Fructose-6-Phosphate Fluorometric Assay Kit	Biovision	Cat# K689
SMI-4a	MedChemExpress	Cat#HY-16576A
Bacterial Strain		
E. coli DH5α	Thermo Fisher	Cat#18258012
E. coli Stable 3	Thermo Fisher	Cat#C737303
E. coli BL21	Thermo Fisher	Cat#C600003

Mouse: BALB/c nude	Nanjing model animal center	N/A
Mouse: C57BL/6- PIM2$^{-/-}$	Cyagen Biosciences	N/A
Experimental Models: Cell Lines		
Human: HEK293T cells	Cell Bank of the Chinese Academy of Sciences	Cat#GNHu17
Human: MCF-7 cells	Cell Bank of the Chinese Academy of Sciences	Cat#TCHu 74
Human: MDA-MB-231 cells	Cell Bank of the Chinese Academy of Sciences	Cat#TCHu227
Oligonucleotides		
See Table S2-S3 for sequences of primers	This paper	N/A
Recombinant DNA		
pCDNA3.0/neo-HA-FBP1	This paper	N/A
pCDNA3.1/neo-Flag-FBP1	This paper	N/A
pCDNA3.0/neo-HA-p65	This paper	N/A
pCDNA3.1/neo-Flag-p65	This paper	N/A
pFlag-CMV-4-PIM2 (WT or K61A)	This paper	N/A
pGEX-4T-1-FBP1	This paper	N/A
pGEX-4T-1-p65	This paper	N/A
PET28a-His-PIM2 (WT or K61A)	This paper	N/A
PET28a-His-FBP1	This paper	N/A
pNF-kB-Luc	This paper	N/A
PGL3-TA-promoter-PD-L1-luc	This paper	N/A
pLVX-IRES-Puro-HA-FBP1 (WT)	This paper	N/A
pLVX-IRES-Puro-HA-FBP1 (S144A)	This paper	N/A
pLVX-IRES-Puro-HA-FBP1 (S144D)	This paper	N/A
pLVX-shRNA1	This paper	N/A
lentiCRISPR v2	Addgene	Cat#52961

Table S2. shRNA and sgRNA sequences used in our research.

shRNA	Sense (5'-3')	Anti-sense (5'-3')
shRNA-Control	TTCTCCGAACGGTCACGT	ACGTGACCGTTCGGAGAA
shRNA-FBP1-1#	CCTTGATGGATCTTCCAACAT	ATGTTGGAAGATCCATCAAGG
shRNA-FBP1-2#	CGACCTGGTTATGAACATGTT	AACATGTTCATAACCAGGTCG
shRNA-PIM2	CCAGTCATTAAAGTCCAGTAT	ATACTGGACTTTAATGACTGG
shRNA-CHIP	TTACACCAACCGGGCCTTG	CAAGGCCCGGTTGGTGTAA
sgRNA-GFP	GGGCGAGGAGCTGTTCACCG	CGGTGAACAGCTCCTCGCCC
sgRNA-p65	GTGACAGTGCGGGACCCATC	GATGGGTCCCGCACTGTCAC

Table S3. The primers used for real-time PCR and CHIP in our research.

**Real-time PCR**		
**Gene**	**Sense (5'-3')**	**Anti-sense (5'-3')**
IL-6	AAAGAGGCACTGGCAGAAAA	TTTCACCAGGCAAGTCTCCT
IL-8	GGTGCAGTTTTGCCAAGGAG	TTTCCTTGGGGTCCAGACAG
MMP2	CGCTCAGATCCGTGGTGAG	TGTCACGTGGCGTCACAGT

VEGF	CTTGCCTTGCTGCTCTAC	TGGCTTGAAGATGTACTCG
β-actin	ATGGCCACGGCTGCTTCCAGC	CATGGTGGTGCCGCCAGACAG
CHIP target		
PD-L1	GGACACCAACACTAGATACCTAAACTG	CTGCCCAAGGCAGCAAAT

Table S4. The details of patient tissues samples

Table S4. The details of patient tissues samples

Number	n
Age	
≤50y	8
>50y	12
Tumor size	
≤2 cm	7
>2 cm	13
TNM stage	
I-II	7
III-IV	13
ER status	
+	11
−	9
PR status	
+	12
−	8
HER2	
+	10
−	10

MATERIALS AND METHODS

Confocal Immunofluorescence Microscopy

The indicated cells were plated on coverslips overnight and fixed in 4% paraformaldehyde, permeabilized with 0.1% Triton X-100. Then they were stained with the indicated primary antibodies overnight at 4°C, followed by incubating with secondary antibodies conjugated with fluorescence in room temperature. Cells were also stained with DAPI. Intracellular localization was visualized using a confocal microscope [1].

Western blotting

Cells were lysed in cell lysis buffer on ice for more than 30 minutes and shaked every 10min, and the lysate was centrifuged at 12000 rpm at 4°C for 15min. 5×loading buffer was added into samples and boiled for 10min after BCA protein quantification. The sample was used for SDS-PAGE analysis and transferred to PVDF membrane. The membrane was blocked with 5% non-fat milk in room temperature for at least 1hr and incubated with indicated primary antibody at 4°C overnight. Then, membrane was incubated with anti-fluorescence secondary antibody for at least 1 hour in room temperature. The protein was visualized by odyssey instrument. The antibodies information is provided in Table S1.

Quantitative RT-PCR

Total RNA was isolated using Total RNA Kit I (Omega Bio-tek). RNA was reversely transcribed using PrimeScript™ RT reagent Kit with gDNA Eraser (Takara) following manufacturer's instructions. Quantitative real-time PCR was performed by TB Green® Fast qPCR Mix (Takara). The result was normalized to β-actin. Sequence information for primers used for qRT-PCR was provided in Table S3.

Dual luciferase reporter assay

Luciferase assays were performed according to the manufacturer's instruction as described previously [1].

Chromatin immunoprecipitation (ChIP) assay

ChIP was performed as described previously [1]. Cell lysate was sonicated and subjected to immunoprecipitation using nonspecific IgG or anti-p65 antibodies. The immunoprecipitated DNA was amplified by real-time PCR following manufacturer's instructions (Milipore). Sequence information for ChIP primers is provided in Table S3.

Measurement of FBP1 enzyme activity

The cell lysates of vector control or expressing FBP1 were added to 100μL reaction buffer. The reaction was incubated at 37°C for 15 min and stopped using deproteinizing sample preparation kit (Biovision). FBP1 catalytic activity was

quantified according to the manufacturer's instruction by using Picoprob fructose-6-phosphate fluorometric assay kit (Biovision).

Cell proliferation analysis

Cell proliferation was assayed using a Cell Counting Kit-8 (CCK-8) kit (MedChemExpress, USA). The indicated cells were seeded in triplicates onto 96-well plates. Following culture for 1, 2 and 3 days, CCK-8 solution was added to each well and incubated at 37°C for 1hr. Then, we used a microplate reader (Multiskan GO, Thermo Scientific, Germany) to determine the optical density at 450nm [1]. All assays were repeated three times.

Clone formation, wound healing assay and cell invasion assay

The indicated cells were plated in 6-well plates at a density of 200-500 cells per well. After the 2-3 week incubation, we removed the medium and stained the cells with crystal violet. The colonies were counted. Wound healing and cell invasion assays were performed as previously described [2]. All assays were repeated three times.

Immunohistochemistry staining

The indicated cells were seeded in six-well plates, which contained coverslips. After 24hr, cells were fixed in 4% paraformaldehyde at room temperature for 10min. Cells were incubated with anti-pS144-FBP1 antibody (1:50). For immunohistochemical analyses, sections were de-waxed, hydrated, and washed. The sections were incubated with 3% H2O2 to block endogenous peroxidase activity after microwave antigen retrieval. Then, the slides were incubated overnight with anti-pS144-FBP1 antibody (1:50) or anti-Ki67 antibody (1:1000), After washed, the sections were then incubated with the horseradish peroxidase-conjugated secondary antibody, and the signals were visualized with diaminobenzidine as the chromogen and counter-stained by hematoxylin.

Generation of Pim1 knockout mice and mouse embryonic fibroblasts (MEFs) cells

PIM2 knockout (PIM2$^{-/-}$) mice was obtained from Cyagen Biosciences (Guangzhou, China). Briefly, PIM2$^{-/-}$ mice lacking exon 3 and 4 of the PIM2 gene were generated using CRISPR/Cas9-mediated genome editing in C57BL/6J embryonic stem cell (gRNA1: GCGCACGCACATCAATTCCATGG; gRNA2: GACATGGGTCCAATGTTCAGAGG). Heterozygous animals were bred to obtain homozygous PIM2$^{-/-}$ mice. MEFs cells were generated from embryonic day 12.5-13.5 embryos of these mice and cultured in DMEM with 15% FBS, 2mM L-glutamine, and 0.1mM MEM nonessential amino acids. All animal experimental procedures were in accordance with the National Institutes of Health Guide for the Care and Use of Laboratory Animals and approved by the Ethical Committee for Animal Experimentation of Weifang medical University.

Reference:

1. Yang T, Ren C, Lu C, Qiao P, Han X, Wang L, Wang D, Lv S, Sun Y & Yu Z (2019) Phosphorylation of HSF1 by PIM2 Induces PD-L1 Expression and Promotes Tumor Growth in Breast Cancer. *Cancer Res* **79**, 5233-5244, doi: 10.1158/0008-5472.CAN-19-0063
0008-5472.CAN-19-0063 [pii].
2. Yang T, Ren C, Qiao P, Han X, Wang L, Lv S, Sun Y, Liu Z, Du Y & Yu Z (2018) PIM2-mediated phosphorylation of hexokinase 2 is critical for tumor growth and paclitaxel resistance in breast cancer. *Oncogene* **37**, 5997-6009, doi: 10.1038/s41388-018-0386-x
10.1038/s41388-018-0386-x [pii].
